# Fertilizer produced from abattoir waste can contribute to phosphorus sustainability, and biofortify crops with minerals

**DOI:** 10.1371/journal.pone.0221647

**Published:** 2019-09-04

**Authors:** Tegan Darch, Robert M. Dunn, Adrian Guy, Jane M. B. Hawkins, Michael Ash, Kwame A. Frimpong, Martin S. A. Blackwell

**Affiliations:** 1 Sustainable Agriculture Sciences department, Rothamsted Research North Wyke, Okehampton, Devon, United Kingdom; 2 Elemental Digest Systems Ltd, Bellinster Business Park, Winkleigh, Devon, United Kingdom; 3 Soil Science Department, School of Agriculture, College of Agriculture and Natural Sciences, University of Cape Coast, PMB, Cape Coast, Ghana; Kyungpook National University, REPUBLIC OF KOREA

## Abstract

Our food security depends on finding a sustainable alternative to rock phosphate for fertilizer production. Furthermore, over 2 billion people worldwide are currently affected by micronutrient deficiencies, and crop concentrations of essential minerals are declining. This paper examines whether a novel multi-element fertilizer, Thallo^®^, can produce crop yields comparable to conventional rock phosphate derived fertilizers, and have an additional benefit of increasing essential mineral concentrations. Thallo^®^, produced from abattoir and recycled industrial by-products, was tested against conventional mineral fertilizers in a pot trial with wheat and grass. In soil, yields were comparable between the fertilizer types, but, in a low-nutrient substrate, Thallo^®^ showed a yield benefit. Elemental concentrations in the plant material typically reflected the relative concentrations in the fertilizer, and Thallo^®^ fertilized plants contained significantly more of some essential elements, such as selenium and zinc. Furthermore, concentrations of the toxic element cadmium were significantly lower in Thallo^®^ fertilized crops. Among the fertilizers, manganese concentrations were greatest in the Thallo^®^, but within the fertilized plants, they were greatest under the mineral fertilizer, showing the complexity of assessing whether nutrients will be taken up by crops. In summary, fertilizers from livestock waste have the potential to improve wheat and grass concentrations of essential elements while maintaining yields.

## Introduction

Phosphorus (P) is essential for life, and is a key limiter of crop yields unless regular applications of fertilizer are used. Currently, much of the P fertilizer used worldwide is derived from rock phosphate ore, but this is both finite, and geographically concentrated in areas such as Morocco and the Western Sahara [1]. Furthermore, rock phosphate can be high in uranium (U) cadmium (Cd), arsenic (As), chromium (Cr), Nickel (Ni) and lead (Pb), toxic elements with no essentiality in plants, animals or people, and the European Commission are considering reducing permissible levels of U and Cd in fertilizers [2]. Alternatives include organic fertilizers, such as manure and slurry, but use of these is declining in more economically developed countries such as Great Britain [3]. This is perhaps due to the increasing physical separation between arable and livestock farms and the cost of transporting organic fertilizers over long distances. It is therefore necessary to find alternative P fertilizer sources, which are sustainable, convenient to farmers, and low in Cd.

An additional consideration is that although conventional nitrogen, phosphorus, potassium, and sulphur (NPKS) fertilizers may contain mineral elements as impurities, concentrations are typically low [4]. A key priority of the UN sustainable development goals [5] is to tackle hidden hunger, where deficiencies of minerals and vitamins in the diet affect health, even where the quantity of food consumed is not severely restricted. Key minerals of concern include iron (Fe), zinc (Zn), selenium (Se), calcium (Ca) and magnesium (Mg) [6], and deficiencies are not limited to less economically developed countries [7–9]. The use of NPKS fertilizers may inadvertently result in lower concentrations of minerals in crops due to a ‘dilution’ effect, because the additional crop yield is greater than the additional uptake of soil minerals [10]. The application of mineral-rich fertilizers to soil, referred to as agronomic biofortification, can increase crop mineral concentrations [4, 11]. However, although a yield benefit can occasionally be demonstrated [10], farmers are unlikely to fertilize crops with trace elements if it is costly to do so, without incentives to encourage it or governmental regulations to require it [12].

Thallo^®^ fertilizer, manufactured by Elemental Digest Systems Ltd (EDS), is derived from abattoir waste supplemented with trace elements from industrial by-products. Abattoir waste includes lairage (bedding material and excreta), gut contents, and parts of the carcass not fit for human consumption (e.g. bones, hooves, tail, and skin), although ‘specified risk materials’ for bovine spongiform encephalopathy (such as the brain and spinal cord) must be disposed of separately. Fertilizers made from bone meal have been tested against conventional fertilizers in the past, usually showing a fertilization rate between that of phosphate rock and super or triple super phosphates [13, 14], but the focus of these types of studies is predominantly on yield or NPK uptake. Furthermore, Thallo^®^ differs from meat and bone meal (MBM) fertilizers because meat trim is removed for human consumption, leaving the bone apatite and organic wastes which are first sterilised and then solubilised via an aggressive chemical reaction, and additional industrial by-products are added to improve the nutrient content. It is unclear whether the trace elements in the Thallo^®^ fertilizer are available to plants, and will significantly increase trace element concentrations in the plant material.

Thallo^®^ fertilizer was compared to mineral fertilizers for wheat and grass growth with the hypotheses: i) plant yields are not affected by the source of fertilizer, ii) Thallo^®^ fertilized crops will have greater essential mineral concentrations but lower Cd concentrations, and iii) essential trace element concentrations in the plant material are not toxic, even when Thallo^®^ is applied at a rate in excess of normal best practice.

## Method

### Manufacture of Thallo^®^

Fresh bone mineral, post meat extraction, is combined with other abattoir organic wastes such as lairage, first stomach content, blood, hoof and horn before being milled to a fine slurry. A metal ion catalyst is added, then the slurry is combined with concentrated sulfuric acid and additional oxidising agents, before undergoing a Department for Environment, Food, and Rural Affairs (Defra) approved Method 1 High Temperature and Pressure Sterilisation Process with the addition of an EDS patented chemical process (International Patent Application Publication No. WO2014202986). The objective of this sterilisation and chemical process is to dissolve and solubilise the animal by-products, for example it hydrolyses starch and cellulose, it converts sulphuric acid to phosphoric acid and calcium sulphate, and organic materials are broken down into smaller, soluble carbon compounds. The resultant acidic mixture is then neutralised using ash from biomass power generation. During the neutralisation process, the insoluble ash compounds are dissolved by the acidic solution, while the heat from the neutralisation process dries the fertilizer material to a damp powder. To this mixture, other industrial by-products with fertilizer value are added to increase the value of Thallo as a marketable product. These by-products can include materials such as reclaimed fertilizer dusts, and waste from the fire extinguisher industry. The resultant powder is then dried, compacted and granulated to form the finished fertilizer.

### Pot trials

Three pot trials were run concurrently, each with a different combination of plant species and planting medium. In the first trial, grass (AberMagic *Lolium perenne*) was grown in a typical non-calcareous pelosol (Hallsworth series), which is a permeable clayey soil, suitable for grassland and livestock rearing. In the second trial, wheat (*Triticum aestivum*) was grown in a typical brown earth (Crediton series), which is a well-drained gritty reddish loamy soil, which is suitable for cereals, roots and some horticultural crops. Typical soil properties can be seen in Blackwell et al [15], and total concentrations are given in Table 1 (analysis method is outlined below). Both soils were taken from sites in South West England, from the 0–10 cm layer, and sieved to <4 mm. In the third trial, grass was grown in acid-washed silica sand substrate, to investigate the potential for use in marginal soils, lacking stores of nutrients and established microbial populations.

**Table 1:** Total element concentrations in the Hallsworth and Crediton series soils, used for the grass and wheat crops respectively. Where available, median total concentrations in European topsoils, as provided by the Forum of European Geological Surveys [16], is given.

	Al	As	Ca	Cd	Co	Cr	Cu	Fe	K	Mg	Mn	Mo	Na	Ni	P	Pb	S	Se	Ti	Zn
Hallsworth series	14900	13.8	1580	0.193	12.4	46.2	24.7	42600	2090	628	761	1.77	210	18.6	1300	33.8	518	0.612	18.8	82.2
Crediton series	11400	11.3	1340	0.146	16.4	41.4	15.6	45800	2150	1220	1290	3.02	135	36.0	643	37.6	201	0.484	120	61.0
FOREGS values		7.30		0.140	8.00	60.0	12.9					0.600		18.0		22.6	230			52.0

In the grass and wheat trials using soil as the growing medium, three fertilizers were tested, each at two application rates, plus there was a nil application control, with all treatments replicated three times. Thus (Nil inputs * 3 replicates) + (3 fertilizer types * 2 application rates * 3 replicates) = 21 pots. In the grass trial that used sand as the growing medium, there was only one application rate of each of the fertilizers, thus (Nil inputs * 3 replicates) + (3 fertilizer treatments * 3 replicates) = 12 pots.

The three fertilizer types were Thallo^®^, a fertilizer containing a slow release N source (Nutralene 40% N, Koch Turf and Ornamental), and a fertilizer containing N as ammonium nitrate (Nitram 34.5% N, CF Fertilizers UK Ltd). The two mineral fertilizers, hereafter referred to as ‘slow release’ and ‘NPK’ respectively, were comprised of individual compounds to approximately match the NPKS levels of the Thallo^®^ fertilizer (6.52% total N, 3.11% acid soluble P, 3.03% water soluble K, and 9.80% S). In addition to the N, the slow release and NPK fertilizers had P as triple super phosphate (46% P_2_O_5_ as Ca(H_2_PO_4_)_2_), K as muriate of potash (60% K_2_O as KCl), and S as Kieserite (50% SO_3_ as MgSO_4_.H_2_O), all manufactured by Origin Fertilizers. Therefore, the slow release and NPK fertilizers differed only in their N source. Fertilizer application rates were either ‘optimal’ or ‘excess’. The optimal levels were based on guidelines in the RB209 Fertilizer Manual for the UK [17], and 60 kg N ha^-1^ was used for the grass trials, and 180 kg N ha^-1^ for the wheat trial. Excess rates of application were twice the optimal levels (120 and 360 kg N ha^-1^ for grass and wheat respectively). The final N:S:K:P ratios of the applied fertilizers were 10 : 13.6 : 4.5 : 3.7 for the slow release and NPK fertilizers, and 10 : 12.7 : 3.3 : 4.1 for Thallo^®^. Analysis of the NPKS in the Thallo^®^ fertilizer, for the purposes of calculating application rates of Thallo^®^ and mineral fertilizers, was performed by Lancrop Laboratories (York, UK) via Leco CNS. All other analyses of P, K, S and trace elements in the fertilizers was analysed using ICP-OES or ICP-MS (depending on the concentration). Fertilizer application rates per pot were calculated based on the surface area of the pot, but fertilizers were evenly incorporated into the soils of each pot prior to sowing seeds.

Plants were grown in 750 g air-dried soil or sand, with either 0.5 g grass seed or 8 wheat seeds. After germination, wheat seedlings were reduced to five, with any ungerminated seeds also removed. Plants were grown in a controlled environment room with a 16/8 hr period of light/dark, and temperatures of 20/16°C respectively. Soil water holding capacity was determined by difference between the mass of saturated soil and gravity-drained soil, and plants were watered to approximately 60% of the water holding capacity (assessed by mass) with an artificial rainwater solution. Plants received the majority of their water from the saucer at their base to encourage deeper rooting. The artificial rainwater solution was a 1 in 1000 dilution of a stock which contained 0.021g Na_3_PO_4_.2H_2_O, 4.562 g CaCl_2_.2H_2_O, 4.05 g MgCl_2_.6H_2_O, 0.091 g FeCl_2_.4H_2_O, 0.562 g NH_4_NO_3_, 1.239 g K_2_SO_4_, 5.843 g NaCl, 0.319 g (NH_4_)_2_SO_4_ and 1.386 g NH_4_Cl dissolved in 1 L milli-Q water. The grass pots were cut at 4 cm above soil height every four weeks for a total of 16 weeks, and wheat plants were grown to maturity (16 weeks).

All harvested plant material was dried on the day of cutting at 85°C for 48 hours, with wheat plants separated into grain, and chaff + straw. Elemental analysis was conducted on the first two cuts of grass grown in sand, on the first three cuts of grass grown in soil, on the wheat grain, and on wheat chaff + straw combined. These samples were milled to a fine powder using a rotary mill, with a subsequent ball milling stage if the end product wasn’t fine enough for chemical analysis, then extracted using a perchloloric acid digest, and analysed using inductively coupled plasma optical emission spectroscopy (ICP-OES) or ICP mass spectrometry (ICP-MS), depending on the elemental concentration [18]. Fertilizer and soil elemental concentrations were measured in the same way, but using an aqua regia digestion.

### Statistical analysis

ANOVA with a nested and crossed treatment structure was used to make several comparisons of interest between the treatments (using Genstat, 18^th^ Edition, VSN International Ltd), with significance assessed as P < 0.05. For analysis of the wheat trial, the treatment structure was Type/Treatment/(Fertilizer*Amount), where Type was Nil or Fertilizer application, Treatment was Nil, Thallo^®^ or Other, Fertilizer was Nil, Thallo^®^, NPK or Slow release, and Amount was Nil, Optimal or Excess. For grass grown in soil, a modified version of this treatment structure was used to include the effect of harvest number, (Type/Treatment/(Fertilizer*Amount))*Cut, with a blocking structure of Unit/Cut, where Unit was the pot number. For grass grown in sand, a simplified version of this treatment structure was used, in order to remove fertilizer amount from the ANOVA. For data from elemental analysis of plant material, separate ANOVAs were run for each of the elements. Non-normal data were transformed before analysis. Due to the structure of the ANOVAs, no post-hoc tests were necessary.

## Results

### Biomass production

**Fig 1:**
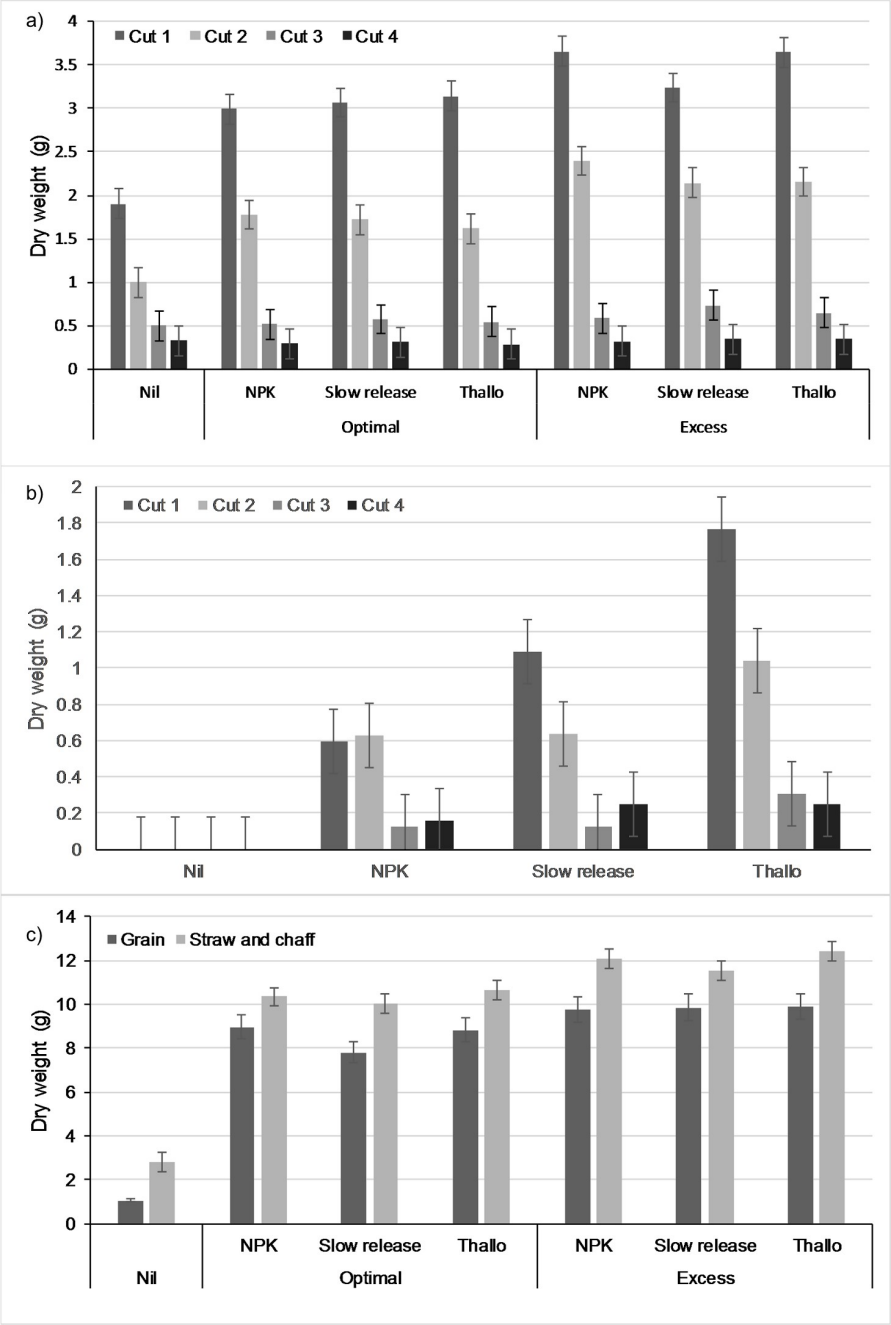
Total biomass production (g dry matter per pot) in the three pot experiments according to the fertilizer treatment and fertilizer application level. a) grass grown in soil across 4 successive cuts, b) grass grown in sand across 4 successive cuts, and c) wheat grain and straw+chaff production. Values are the mean of 3 replicates, and their standard error.

The grass sward in both sand and soil experiments was cut four times, and the biomass decreased (p < 0.001) over time across all treatments (Fig 1A and 1B). The trend in the sand-grown Nil pots differed due to poor biomass production, with a mean of 0.002 g DM/pot in the first cut, and no subsequent biomass production. Fertilizer addition (either Thallo^®^, NPK or slow release) resulted in greater biomass production (p < 0.001) compared to the Nil treatment (Fig 1A and 1B), in both the sand- and soil-grown grass experiments. In both experiments, there was also a significant interaction with cut (p < 0.001), where the difference between the fertilized and Nil treatments decreased with cut number. For example, in cut 1 in the soil-grown grass, the Nil pots contained a mean of 1.90 g DM/pot, with a mean of between 2.99–3.66 g DM/pot in the fertilized treatments, while in cut 4, the Nil pots and fertilized treatments contained 0.33 g and 0.29–0.35 g DM/pot respectively. The aboveground wheat biomass was separated into grain, and straw+chaff, and both showed a significant increase in biomass in the pots with fertilizer addition, compared with the Nil treatment pots (Fig 1C). Wheat grain increased from a mean of 1.1 g DM/pot in the Nil treatment, to a mean of between 7.8–9.0 g in the fertilized treatments at optimal application levels, and increased from 2.8 g to 10.0–10.6 g for straw+chaff.

Two rates of fertilizer application were tested in the soil-grown grass and the wheat experiments, and the biomass of the grass, wheat grain, and wheat straw+chaff were all greater (p < 0.001) at the excess fertilizer application rate compared with the optimal rate (Fig 1A and 1C). In the soil-grown grass experiment, there was also a significant interaction between fertilizer application rate and cut number (p < 0.001). At cut 1, optimal fertilizer levels produced a mean of between 2.99 and 3.14 g DM/pot, while excess fertilizer levels resulted in a mean of 3.24–3.66 g DM/pot. But at cut 4, optimal and excess fertilizer levels gave 0.29–0.32 and 0.33–0.35 g DM/pot respectively. Comparing the biomass production in the Thallo^®^ fertilized treatments to the other fertilized treatments (NPK or slow release) showed variable effects across the experiments. There was no significant effect on the biomass of soil-grown grass (p = 0.918), and no significant interaction with cut (p = 0.076), and there was no significant difference in wheat grain biomass (p = 0.244). However, there was an increase in wheat straw+chaff biomass of up to 7.5% with Thallo^®^ fertilizer (p = 0.01), and there was an increase in the biomass of sand-grown grass (p < 0.001), and there was also a significant interaction with cut (p < 0.001). In cut 1, Thallo^®^ fertilized pots had a mean yield of 1.76 g DM/pot, whereas the NPK and slow release fertilizers had an of 0.60 and 1.09 g DM/pot respectively. By cut 4, yields had reduced to 0.25 g DM/pot for Thallo^®^ fertilized grass, and 0.16 and 0.25 g DM/pot for the NPK and slow release fertilized grass.

### Elemental composition

Plant material was analysed for 20 elements. Concentration data for each treatment, within each growth experiment and sample type, and across each cut (in the case of grass) are available in S1–S4 Tables. Significance P values are summarised in Table 2, and indicate whether concentrations increased or decreased due to the treatment.

**Table 2:** Significance P values of i) adding fertilizer (Nil v fertilized with the 3 fertilizers combined), ii) the fertilizer application rate (optimal v excess), and iii) the cut number (over 2 curs for sand-grown grass, or 3 cuts for soil-grown grass), for each of the elements analysed by ICP-OES or ICP-MS. Data is shown for each of the growth experiments (soil-grown grass, sand-grown grass, wheat grain, and wheat straw+chaff), and interaction of the effect with cut number is given for grass experiments.

	i) Nil v fertilized †				ii) Optimal v excess ‡				iii) Cut § ¶	
	Grass in soil	Grass: interaction with cut	Wheat grain	Wheat straw+chaff	Grass in soil	Grass: interaction with cut	Wheat grain	Wheat straw+chaff	Grass in sand	Grass in soil
Al	0.335	0.604	<0.001^NIL^	<0.001^NIL^	0.469	0.29	0.339	0.552	0.049^INC^	<0.001^VAR^
As	0.383	0.053	0.125	0.928	0.78	0.029	0.975	0.891		<0.001^INC^
Ca	0.235	0.002	0.003^NIL^	<0.001^FERT^	0.213	0.025	<0.001^VAR^	<0.001^VAR^	<0.001^INC^	<0.001^INC^
Cd	0.428	0.098	<0.001^FERT^	<0.001^FERT^	0.373	0.766	0.635	0.611		<0.001^VAR^
Co	0.424		<0.001^NIL^		0.017^VAR^		0.01^VAR^			
Cr	0.058	0.415	<0.001^NIL^	0.012^NIL^	0.124	0.178	0.599	0.4	0.003^INC^	<0.001^INC^
Cu	0.477	0.076	<0.001^NIL^	0.325	0.078	0.84	0.01^EXCESS^	<0.001^EXCESS^	<0.001^DEC^	<0.001^VAR^
Fe	0.012^NIL^	0.105	<0.001^NIL^	<0.001^NIL^	0.449	0.742	0.167	0.492	0.937	<0.001^VAR^
K	0.147	<0.001	0.004^NIL^	0.003^FERT^	0.544	0.003	<0.001^EXCESS^	<0.001^EXCESS^	<0.001^DEC^	<0.001^VAR^
Mg	<0.001^FERT^	0.008	<0.001^NIL^	<0.001^FERT^	<0.001^EXCESS^	0.05	<0.001^EXCESS^	<0.001^EXCESS^	0.003^INC^	<0.001^DEC^
Mn	<0.001^FERT^	<0.001	<0.001^NIL^	0.062	0.199	0.028	0.09	0.053	0.027^INC^	<0.001^DEC^
Mo	0.177	*	<0.001^NIL^		<0.001^OPT^		0.113			
Na	0.466	0.047	0.002^NIL^	0.001^FERT^	0.277	0.052	0.005^EXCESS^	<0.001^EXCESS^	<0.001^INC^	<0.001^VAR^
Ni	0.003^NIL^	0.809	<0.001^NIL^	0.044^NIL^	0.033^VAR^	0.334	0.427	0.58	<0.001^INC^	<0.001^VAR^
P	0.062	<0.001	<0.001^NIL^	0.012^FERT^	<0.001^EXCESS^	<0.001	<0.001^EXCESS^	<0.001^EXCESS^	0.009^DEC^	<0.001^DEC^
Pb			0.003^NIL^	0.004^NIL^			0.661	0.994		
S	<0.001^FERT^	<0.001	0.601	<0.001^FERT^	0.026^EXCESS^	0.002	<0.001^EXCESS^	<0.001^EXCESS^	0.029^INC^	<0.001^DEC^
Se	0.036^NIL^		N/A		0.038^VAR^		0.018^EXCESS^			
Ti	0.744		<0.001^NIL^	<0.001^NIL^	0.011^VAR^		0.09	0.87		
Zn	0.246	0.592	<0.001^NIL^	<0.001^FERT^	0.306	0.556	<0.001^EXCESS^	<0.001^EXCESS^	<0.001^DEC^	<0.001^VAR^

* Gaps in table are because either element was below the limit of detection, or because only 1 cut was analysed, and therefore interaction with cut cannot be tested.

^†^ In the Nil v fertilized comparison, the superscript indicates whether NIL or FERT (fertilizer applied) pots had significantly greater concentrations of the element in the crop (P < 0.05).

^‡^ In the optimal v excess comparison, the superscript indicates whether OPT (optimal) or EXCESS fertilizer application levels resulted in significantly greater concentrations of the element (P < 0.05) in the crop. The subscript VAR indicates that whether optimal or excess fertilizer led to significantly greater element concentrations varied between fertilizer types.

^§^ In sand, 2 herbage cuts were analysed. In soil, 3 herbage cuts were analysed.

^¶^ In the cut comparison, the superscript indicates whether element concentrations either significantly (P < 0.05) INC (increased) or DEC (decreased) with cut number, or whether the effect was VAR (variable, inconsistent pattern across cuts).

A comparison of the elemental composition of plants in the Nil versus those in the fertilized pots (Thallo^®^, NPK and slow release) could not be made for the grass grown in sand, due to the very small quantity of herbage in this experiment. However, across the other experiments and sample types (grass grown in soil, wheat grain, and wheat straw+chaff), and across the elements measured, 67% of the P values comparing element concentrations crops in Nil fertilizer and fertilizer applied pots (Table 2, Nil v fertilized comparison) showed a significant difference. Where a significant effect was found, the elemental composition of plants in the Nil treatment was greater than in the fertilized treatments for 68% of those P values. The effect of fertilizer application was not always consistent across experiments, as, for example, manganese (Mn) concentrations in soil grown grass were greatest in fertilized treatments, but in wheat they were greatest for Nil treatments. Similarly, the effect of fertilizer treatment was not always consistent across the wheat material, with greater P concentrations in grain under the Nil treatment, but greater in the straw+chaff under the fertilized treatments.

A similar comparison to examine the effect of fertilizer level on element concentrations in plant material showed 46% of p values, across experiments, sample types and elements, to be significant (Table 2, optimal v excess comparison). Of these, optimal fertilizer levels led to greater element concentrations on only one occasion, for molybdenum (Mo) in soil grown grass. For a further 18 p values (69% of total significant p values), excess fertilizer led to greater element concentrations. However, for 7 p values (27% of total significant p values), the type of fertilizer (NPK, slow release or Thallo^®^) affected whether the optimal or excess fertilized treatments had the greatest element concentrations.

**Fig 2:**
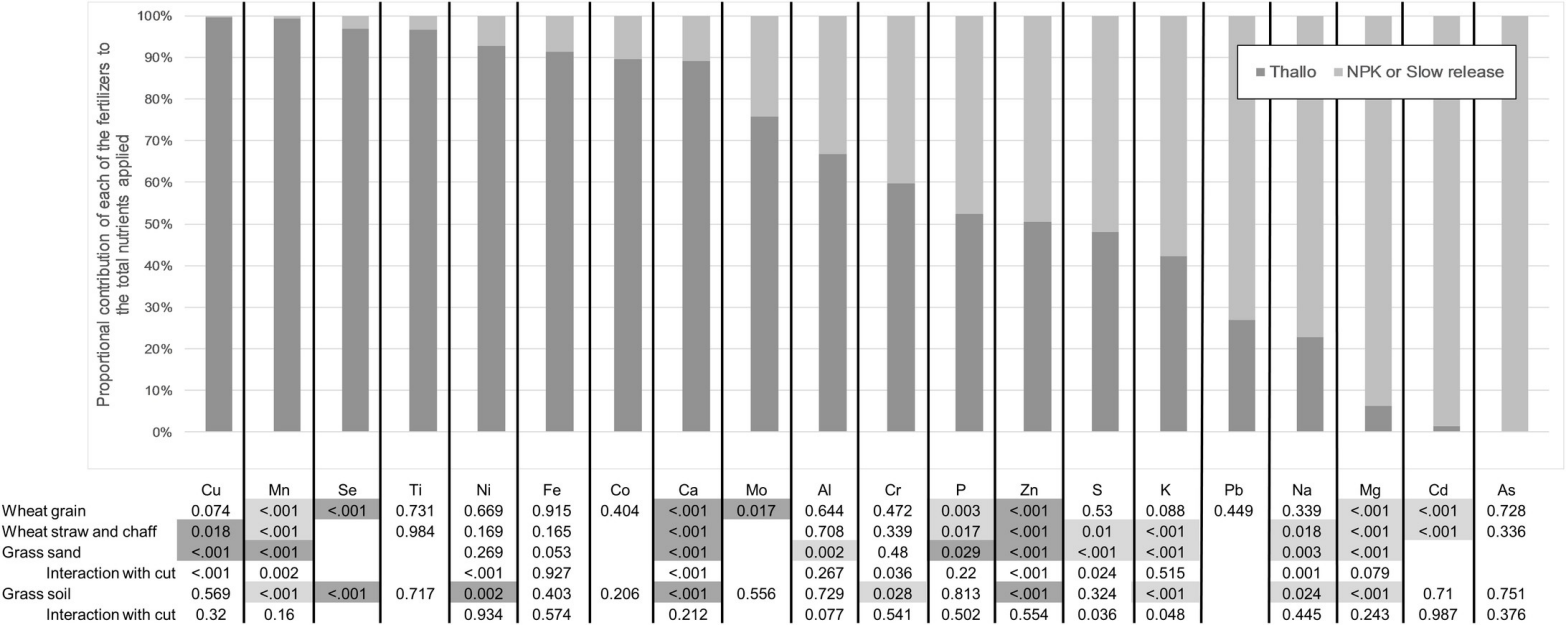
Relative additions of each element to the soil in Thallo^®^ and mineral fertilized treatments, and statistical effects of the fertilizer type on elemental concentrations in plant material. The bars indicate the relative contribution of each of the Thallo^®^ and mineral fertilizers (NPK or slow release) to a sum of the total quantity of each element applied to the pots. Statistically significant effects of fertilizer type on elemental composition of plants are coloured light grey to indicate that mineral fertilized plants have the greatest element concentration, or dark grey to indicate that Thallo^®^ fertilised plant concentrations are greatest.

54% of the P values comparing fertilizer type (Thallo^®^ or mineral fertilizers), across the experiments, sample types and elements measured, had a significant value (Fig 2). When any significant effects on elements that could be considered contaminants (As, Cd, Cr, [Ni and Pb) were discounted, the remaining significant differences in element concentrations between the fertilizer types showed greater values in the mineral fertilized plants for 55% of p values. The effect of fertilizer type on the concentration of any given micronutrient was generally consistent between wheat grain and straw+chaff, and between experiments. The plant material from mineral fertilized pots contained greater concentrations of Cd in wheat grain and wheat straw+chaff (p < 0.001), and Cr in soil-grown grass (p = 0.028), than the plant material from Thallo^®^ fertilized pots. In contrast, Ni concentrations in soil grown grass were significantly greater (p = 0.002) in Thallo^®^ compared with mineral fertilized pots. There were no significant effects of fertilizer type on the herbage content of As or Pb.

Grass experiments were cut on several occasions, and elemental composition measured in the first two cuts in sand grown grass, and the first three cuts in soil grown grass. There was almost always a significant effect (p ≤ 0.049) of cut number on the elemental composition of the plant material (96% of the P values across the two experiments, and across elements; Table 2, cut comparison). However, the effect of cut number on elemental composition was not consistent, either between or within experiments. In sand-grown grass, 67% of significant P values were due to element concentrations increasing with cut number. In soil-grown grass, increasing cut number resulted in an increase in element concentration in 20% of the significant P values, a decrease in 27% of the significant P values, and showed no clear trend (for example, because the third cut was intermediate of the first and second cuts) in 53% of the significant P values.

### Mineral concentrations relative to fertilizer composition

The quantity of mineral fertilizers used were such that the quantity of NPKS applied to each pot was equivalent to the Thallo^®^, and the mineral fertilizers differed from one another only in their N source. The fertilizers used to match the PKS levels to Thallo^®^ contained some trace elements, and the total application of each element for each fertilizer are given in Table 3. There was no difference between the trace element composition of the two N sources, and so the mineral fertilizers can be considered the same in terms of their trace element application to the soils, and therefore the reference to mineral fertilizers here is to either the NPK or slow release fertilizer.

**Table 3:** Total mass of each element applied per pot for each of the three fertilizer types–NPK, slow release, and Thallo^^®^^. Application rates are for the optimal fertilizer application rate for grass; excess fertilizer application rates for grass resulted in the element mass applied being twice the values in the table, and optimal and excess fertilizer application rates for wheat resulted in the element mass applied being three and six times the element masses shown in the table, respectively.

	Al	As	Ca	Cd	Co	Cr	Cu	Fe	K	Mg	Mn	Mo	Na	Ni	P	Pb	S	Se	Ti	Zn
	mg pot^-1^																			
NPK	0.58	0.00099	22.3	0.00443	0.0001	0.038	0.001	0.54	32.9	72.0	0.007	0.00083	7.50	0.002	27.0	0.00062	100.3	0.0002	0.0094	0.035
Slow release	0.58	0.00094	22.3	0.00443	0.0001	0.038	0.001	0.54	32.9	71.7	0.007	0.00083	7.51	0.002	27.4	0.00057	100.3	0.0002	0.0093	0.036
Thallo	1.17	0.00000	180.3	0.00007	0.0011	0.056	0.244	5.61	24.1	4.8	0.901	0.00259	2.21	0.028	30.2	0.00021	93.2	0.0077	0.2718	0.037

Fig 2 shows the relative additions of individual elements by either the Thallo fertilizer or the mineral fertilizer (N.B. only one mineral fertilizer formulation is used because the slow release and NPK fertilizers varied only in the form of N added, and all other components were the same, as shown in Table 3). The proportional contributions were derived by calculating the quantity of each element in each of the Thallo and mineral fertilizers (as shown in Table 3), summing that quantity (e.g. quantity of element in Thallo fertilizer applied + quantity of element in mineral fertilizer applied), and then calculating the ratio of that element contained in each fertilizer dose as a percentage of the total. The figure shows that mineral fertilizers provided more K, Pb, sodium (Na), Mg, Cd, and As to the soil than Thallo^®^. Also, fertilizer type had a significant, though complex, effect on element concentrations in plant material. For instance, elements that were at a greater concentration in mineral fertilizers were also at a greater concentration in mineral-fertilized plants. Furthermore, although the different fertilizer types contained equal amounts of P, S, and Zn, the elemental composition of the plant material was, in many cases, significantly affected by fertilizer type. For example, Thallo^®^ fertilized plants always contained more Zn than mineral fertilized plants, but, where a significant difference between the plants was measured for S, concentrations were greater in the mineral fertilized plants. Concentrations of P in the plant material were dependent on both fertilizer and crop type, with wheat containing greater P concentrations under mineral fertilizers, whereas grass had a greater P concentration under the Thallo^®^. Concentrations of Cr, aluminium (Al), Mo, Ca, cobalt (Co), Fe, Ni, titanium (Ti), Se, Mn, and copper (Cu), were greater in the Thallo^®^ fertilizer compared with the mineral fertilizers, and this was generally reflected in the concentrations of these elements in the plant material. However, despite Thallo^®^ containing slightly more Al and Cr than the mineral fertilizers, Al concentration in sand-grown grass and Cr concentration in soil-grown grass were greater under the mineral fertilizer. Moreover, the greatest exception was Mn, where 0.901 g/pot was applied in Thallo^®^ fertilized pots, and 0.007 g/pot in mineral fertilized pots. Despite this, mineral fertilized plants had greater concentrations of Mn in wheat grain, wheat straw+chaff, and soil-grown grass. Only sand-grown grass had the greatest Mn concentration under Thallo^®^ fertilizer.

## Discussion

### Thallo^®^ is a sustainable alternative to conventional fertilizers

Every year, the European Union produces over 20 million tons of animal by-products, which are the parts of animals not consumed by people [19]. Currently abattoir waste can be recycled to land in the form of meat and bone meal (MBM) fertilizers, which are produced from pressure sterilizing animal carcasses to reduce the risk of disease transmission [20]. However, the plant availability of P in these fertilizers is low, typically 50% in the first year after application [21], and therefore yield from these fertilizers tends to be lower than from single or triple superphosphate [13, 14]. In contrast, this study showed Thallo^®^ to have an equal or greater biomass production compared to the mineral fertilizers. The Thallo^®^ production process means that even though the fertilizer has been sterilised, it cannot be considered a MBM fertilizer. No meat remains on the bones due to its removal for beef stock production, and the pressure sterilization process includes additions of concentrated sulphuric acid, catalysts, and an oxidising-agent, and this is neutralised with ash containing calcium oxide. The resulting dicalcium phosphate is considerably more soluble (-log Ksp of 6.6) than the calcium hydroxyapatite found in animal bones (-log Ksp of 58.4, with greater numbers representing a decreased solubility, on a logarithmic scale) as used in MBM fertilizers. Furthermore, Thallo^®^ also contains P from other waste streams, such as fertilizer dusts and fire extinguisher waste material. The comparable yield production of Thallo^®^ fertilized plants with the mineral fertilized plants indicates that the P in the two fertilizer types is likely to be similar. These initial data indicate that even in the short term, the recycled nature of Thallo^®^ fertilizer means that it can be considered as a sustainable alternative to conventional fertilizers.

Crop yields may be increased not only by NPK fertilizers, but also due to micronutrient additions. In a review of 26 studies, Dimkpa and Bindraban [10] found that when either a single micronutrient or a combination of two micronutrients were used in addition to NPK fertilizers, there was a median yield increase of 21% relative to just NPK fertilizer use. In our experiments, sand-grown grass had a significantly greater yield when fertilized with Thallo^®^ than with either of the mineral fertilizers. Without further data it is not possible to determine whether the difference was because the Thallo^®^ had greater concentrations of essential micronutrients than the mineral fertilizers, whether Thallo^®^ had greater availability of NPKS or micronutrients, or whether there is another factor of importance. Despite the difference in yield between the Thallo^®^ and mineral fertilizers in sand-grown grass, there was no effect on the biomass of wheat grain or soil-grown grass. The silica sand was effectively an inert substrate, and, unlike soil, could not act as a reservoir of elements, and consequently the effects of additional trace elements were more dilute in the soil experiments. The soils used to grow the wheat and grass were both from SW England, where the trace element concentrations are either at or above the median concentrations for European topsoils (Table 1). Therefore, it may be that in other parts of Europe, or in arable soils where trace element concentrations have been depleted, that the trace elements in Thallo^®^ could have a significant effect on yield.

### Crop mineral concentrations generally reflect fertilizer mineral concentrations, but there can be trade-offs in a multi-element fertilizer

Crop yields are important, but it is also vital to produce high quality food. One component of this is that the crops contain sufficient concentrations of essential trace elements, while concentrations of potentially toxic elements in the plant material are minimised [5]. Over time, there have been reductions in the trace element concentration of crops, which is likely to be due to the breeding of higher-yielding plant cultivars, but may also be due to the reduction in trace element concentrations in soil [8, 9]. We found that the crops (wheat, soil-grown grass) in the fertilized treatments (Thallo^®^, NPK and slow release combined) only had a significantly different element concentration to the Nil treatment two-thirds of the time. Of these differences, two-thirds were caused by element dilution due to fertilizer application, the other third by an increase in the element concentration in the plant material. It is expected that the use of NPK fertilizers can result in decreased concentrations of trace elements within the crop, termed dilution, possibly because the plant grows faster than the trace elements are mobilised or made bioavailable in the rhizosphere [10, 22]. However, some studies find no dilution or increased concentrations of elements, or very few effects, in cereal grain due to long-term fertilizer applications [23, 24]. In a review, Rietra et al. [25] found that dilution of trace elements due to N or P fertilizer application was most likely if either the soil was low in trace elements, which the soils from this study were not particularly (Table 1), or there was preferential uptake of N or P over the trace element in question. Our data show that whether an effect of fertilizer application is seen, and whether element dilution or an increase in concentration occurs, is complex.

Our results showed significant differences between the Thallo^®^ and mineral fertilizers in the concentration of many elements, most notably Cu, Mn, Se, Ca, P, Zn, S, K, Na, Mg, and Cd. Generally, significant differences in any given crop resulted from greater concentrations of that element in the fertilizer applied. Using element specific fertilizers has been shown to increase the concentrations of Se, Zn, I, Mn, Mo, Co and Cu in crops [11, 26, 27], but this study shows that the elements do not have to be added individually, or added in a form that is specifically designed to be plant available, in order to increase plant concentrations of that element.

However, there were elements that did not follow the trend described. One example from our results is Mn, where concentrations were much greater in Thallo^®^ fertilizers than in the mineral fertilizers, yet plant concentrations (with the exception of sand-grown grass) were much greater when fertilized with either of the mineral fertilizers. Furthermore, Zn concentrations in the Thallo^®^ and mineral fertilizers were identical, yet Zn concentrations in plant material were significantly greater when grown with the Thallo^®^ fertilizer. Reasons for this could include potential differences between the fertilizers in the bioavailability of the elements, or differences between the fertilizers causing differences to soil pH, and hence element availability [26]. Leaching of elements during these experiments did not occur, as they were watered from the base of the soil. However, plant uptake of trace elements is not only affected by soil concentrations and the bioavailability of that element, but by other elements in the soil, the interaction of which may be positive (synergism) or negative (antagonism). Experimental trials have shown Zn to have antagonistic interactions with P, Ca, Cu, Mg and K [22, 25, 28]. Since Zn concentrations were greater in Thallo^®^ fertilized plants, it is reasonable to expect that these antagonistic elements would be at a greater concentration in the mineral fertilized plants. However, only P concentrations (in wheat), and Mg and K concentrations (in wheat and grass), were, while Ca and Cu were greater under the Thallo^®^ fertilizer. Although Cd is known to be positively correlated with Zn, as well as Fe and Cu [29], synergism between these elements was not seen in our experiments. Similarly, Mn is known to be inhibited by Ca, Mg and Zn [25], but our results show Mn and Mg concentrations to be greatest under the mineral fertilizers, and Ca and Zn, to be greatest under the Thallo^®^ fertilizers.

These results indicate that our understanding of element synergism or antagonism, gained from controlled growth trials, are not able to fully describe the complexity of data gained in a multi-element trial such as this. Furthermore, it indicates that a multi-element fertilizer such as Thallo^®^ will always have trade-offs for plant nutrient concentrations, as the presence of some trace elements may have antagonistic effects on others.

### A single fertilizer application was usually insufficient to affect whether crop concentrations were sufficient, deficient, or toxic relative to human and livestock requirements

Due to the necessity of inducing a deficiency of an element in humans or animals before a threshold requirement can be set, trace element requirements are not clearly defined. Furthermore, the variety of foodstuffs in human, and to some extent livestock, diets, mean that reference intakes are often set in values per day, rather than as a concentration in each food item. This means that estimation of crop quality in terms of mineral content for human or livestock health are hard to determine. However, Table 4 gives some estimated reference intake values as concentrations for mono-gastric and ruminant animals [30], and mineral concentrations known to be toxic to plants [26].Also shown in Table 4 are the ranges of mineral concentrations measured in our crops, and these have been colour-coded to show whether they are sufficient, deficient, or toxic relative to requirements. We have assumed that the crop (whether wheat for human consumption, or grass for ruminant consumption) comprises the entirety of the diet, in order that the sufficiency or deficiency concentrations can be compared directly with the measured concentrations.

**Table 4:** Literature data for the range of element concentrations considered sufficient or toxic for monogastrics (used to represent human health), ruminants, and plants. Also presented are the range of concentrations in plant material in our experiments, separated by experiment and fertilizer treatment.

	Sufficiency range		Toxicity range	Wheat grain			Soil-grown grass			Sand-grown grass	
	Monogastrics	Ruminants	Plants	Nil	Thallo	Mineral	Nil	Thallo	Mineral	Thallo	Mineral
						mg kg^-1^ DM					
Cu	3–8	9–11	20–100	9.3	3.7–5.1	3.4–4.3	4.5–7.8	4.6–8.9	4.2–9.6	10–13	1.2–2.1
Fe	40–100	13–50	>1000	1303	29–49	28–49	41–743	48–491	29–513	57–64	93–191
Mn	2–60	15–40	300–500	146	63–75	80–99	135–328	102–394	140–527	50–108	27–66
Mo	<0.2	<0.2	10–50	1.4	0.039–0.053	0.001–0.023	0.25	0.078–0.36	0.14–0.26		
Zn	50–100	20–55	100–400	73	55–86	48–58	26–32	28–38	25–36	18–31	5.4–7.1
Ni	0.05–0.2	0.3–0.5	10–100	11	0.76–0.93	0.76–0.98	5.7–9.7	4.8–9.6	3.7–8.1	1.4–3.9	1.8–3.9
Co		0.1–0.2	15–50	0.34	0.0002–0.001	0.0002–0.011	0.086	0.042–0.089	0.045–0.10		
Se	0.15–0.3	0.1–0.3			0.47–0.66	0–0.22	0.52	0.70–0.80	0.038–0.43		

Comparison of trace element concentrations in wheat grain with the requirements of monogastrics (used as a proxy for human health), and in sand- and soil-grown grass with requirements for ruminants indicated that the fertilized crops generally contained sufficient trace element concentrations (Table 4). The main exceptions were Cu and Co concentrations in soil-grown grass, which were deficient. Although our results found some effect of fertilizer application rates and the type of fertilizer (Thallo^®^ or mineral) on crop elemental concentrations, typically this was not sufficient to affect whether crops were deemed sufficient or deficient in an essential element. The exception was Se, which was sufficient in Thallo^®^ fertilized crops, but deficient under the mineral fertilizers. However, only a single application of fertilizer was tested in a short-term study, and accumulation of elements in soil with repeated applications may increase differences between fertilizer types.

When considering the toxicity of elements, it is important to distinguish between essential elements accumulated in plant material at too great a concentration, and concentrations of non-essential elements (e.g. As, Cd, Pb). Concentrations of non-essential elements in our crops were an order of magnitude lower than concentrations permitted in animal feed by the European Commission, of 2.7, 11 and 1.1 mg kg^-1^ DM for As, Pb and Cd respectively [31]. However, the non-essentiality of these elements, and the potential for accumulation in the body of elements such as Cd [32], means we need to minimise applications to the soil and uptake by crops. Thallo^®^ fertilized wheat plants had significantly lower concentrations of Cd in the grain and in the straw+chaff than in the mineral fertilized plants. The source of rock phosphate from which the mineral fertilizers are derived is important in determining the concentration of Cd in the fertilizer [32], whereas the Thallo^®^ fertilizer is derived from consistently low Cd sources such as abattoir waste. Therefore, using Thallo^®^ instead of an enriched Cd rock phosphate derived P source, can be an important mechanism for decreasing Cd concentrations in plants.

A few of the essential elements were also present in the plant material at potentially toxic concentrations, i.e. above the minimum value in the toxicity range in Table 4, but with the caveat that the plant toxicity values are not specific to the crops used in this study. The toxicity of the essential elements is split into ‘plant’ and ‘ruminant’ in Table 4. Plant toxicity relates to the effects on the plant itself, and greater levels of an element may result in reduced crop yield. However, regardless of whether concentrations of an element are toxic to the crop itself, they may be toxic to the species that consumes them, and again result in a range of deleterious effects, including reduced weight gain. In wheat grain, toxicities of Fe and Ni are of little concern as they were only in the Nil treatment, and it is uncommon to grow wheat without fertilizer application. In soil-grown grass, the greatest concentrations of Mn were potentially toxic to the plant, and this occurred across the Nil, Thallo^®^, and mineral fertilized treatments, and regardless of the rate of fertilizer application. This indicates that it is an, as yet unidentified, factor of the soil used for the grass experiment, and may indicate why no similar toxicity was measured in the wheat grain, which was grown on a different soil type. Furthermore, toxicity values for ruminants were not available, so the toxicity values are for plants of an unspecified species [26]. As no toxicity symptoms were noted in our plants, it is possible that Mn was not actually toxic to grass at the concentrations measured in our study. In sand-grown grass, Thallo^®^ fertilized plants had potentially toxic concentrations of Cu, whereas under the mineral fertilizer, concentrations were within acceptable limits. However, there was no significant difference between the mineral and Thallo^®^ fertilizers in Cu concentrations for wheat grain or soil-grown grass. Therefore, it is probable that Cu toxicity would not be an issue in field grown crops, but this needs further investigation, in order to help contribute to a better formulation of the fertilizer.

### Future research requirements

While we acknowledge the limitations of a pot study to investigate the effects of mineral and micronutrient-enhanced fertilizers on plant concentrations of nutrients, this study demonstrated intriguing relationships between the elemental composition of fertilizers, the elements themselves, and their uptake by plants. Clearly, the complex interactions of the elemental composition of fertilizers and the factors influencing plant uptake rate and concentration of those elements were not resolved at the scale of the study reported here. Indeed, the variation in element concentrations in plants may be further influenced by soil and crop type, and by seasonal and inter-annual drivers. Therefore, we suggest that longer-term field-scale trials are required to test the agronomic efficiency and the biofortification potential of micronutrient-enhanced fertilizers such as Thallo^®^, that have been produced from recycled abattoir and industry by-products. We also suggest that additional information on optimal concentrations of micronutrients in grains for human consumption would be advantageous. Currently, recommended intakes for the human population are often given as daily requirements [33], rather than concentrations, due to the varied range of foodstuffs that comprise the diet. However, as wholegrain cereals remains a major source of many micronutrients [33], target concentrations in the grain would be beneficial for both agronomists and plant breeders.

## Supporting information

S1 Table Mean element concentrations in sand grown grass, for each of the three fertilizer types (NPK, Slow release, and Thallo^^®^^), and for both of the first two herbage cuts. Values in parentheses are the lower and upper confidence intervals, calculated at 95%. Only elements with measured concentrations above the limit of detection are shown.
(DOCX)

S2 Table Mean element concentrations in soil grown grass, for each of the three fertilizer types, for each of the fertilizer application levels (nil, optimal, and excess), for S2A Table: Cut 1, S2B Table: Cut 2, and S2C Table: Cut 3. Values in parentheses are the lower and upper confidence intervals, calculated at 95%. Only elements with measured concentrations above the limit of detection are shown, therefore for Co, Mo, Se, and Ti there are only data for the first cut.
(DOCX)

S3 Table Mean element concentrations in wheat grain, for each of the three fertilizer types, and for each of the fertilizer application levels. Values in parentheses are the lower and upper confidence intervals, calculated at 95%. Only elements with measured concentrations above the limit of detection are shown.
(DOCX)

S4 Table Mean element concentrations in the combined wheat straw and chaff, for each of the three fertilizer types, and for each of the fertilizer application levels. Values in parentheses are the lower and upper confidence intervals, calculated at 95%. Only elements with measured concentrations above the limit of detection are shown.
(DOCX)
