# Supplementary material for: Fertilizer produced from abattoir waste can contribute to phosphorus sustainability, and biofortify crops with minerals
Source: PLoS One. 2019 Sep 4;14(9):e0221647. doi: 10.1371/journal.pone.0221647 (PMC6726140; doi:10.1371/journal.pone.0221647)
Supplement: S1 Table — Values in parentheses are the lower and upper confidence intervals, calculated at 95%. Only elements with measured concentrations above the limit of detection are shown. (DOCX) [file pone.0221647.s001.docx]

|  | Cut 1 | | |  | Cut 2 | | |
| --- | --- | --- | --- | --- | --- | --- | --- |
|  | NPK | Slow release | Thallo |  | NPK | Slow release | Thallo |
|  | mg/kg DM | | |  | mg/kg DM | | |
| Al | 36 (22.8, 57.9) | 24 ((14.9, 38.0) | 17 (10.5, 26,8) |  | 46 (29.0, 73.8) | 69 (43.4, 110) | 19 (12.1, 30.9) |
| Cr | 2.1 (1.25, 3.64) | 1.1 (0.62, 1.80) | 1.1 (0.62, 1.79) |  | 2.0 (1.16, 3.37) | 3.2 (1.88, 5.48) | 5.0 (2.95, 8.59) |
| Cu | 2.1 (1.73, 2.48) | 2.1 (1.76, 2.51) | 13 (12.3, 13.0) |  | 1.2 (0.80, 1.55) | 1.4 (1.05, 1.80) | 10 (9.81, 10.6) |
| Fe | 170 (88.1, 256) | 110 (27.1, 195) | 57 (0.00, 141) |  | 93 (9.11, 177) | 190 (107, 275) | 64 (0.00, 148) |
| Mn | 66 (51.6, 81.2) | 27 (12.3, 41.9) | 50 (34.9, 64.5) |  | 57 (42.6, 72.2) | 28 (12.8, 42.4) | 110 (93.4, 123) |
| Ni | 3.9 (2.99, 5.00) | 1.8 (1.40, 2.35) | 1.4 (1.10, 1.84) |  | 3.0 (2.35, 3.92) | 2.6 (2.01, 3.36) | 3.9 (3.01, 5.03) |
| Zn | 6.9 (5.58, 8.20) | 7.0 (5.66, 8.28) | 32 (30.2, 32.8) |  | 5.4 (4.12, 6.74) | 7.1 (5.82, 8.44) | 18 (16.5, 19.1) |
|  | g/kg DM | | |  | g/kg DM | | |
| Ca | 1.7 (1.19, 2.31) | 1.4 (0.80, 1.93) | 6.8 (6.23, 7.35) |  | 1.3 (0.72, 1.85) | 2.0 (1.47, 2.59) | 15 (14.2, 15.3) |
| K | 36 (31.2, 40.4) | 26 (21.8, 31.0) | 14 (9.48, 18.7) |  | 24 (19.4, 28.7) | 17 (12.5, 21.8) | 5.4 (0.81, 10.0) |
| Mg | 6.3 (4.92, 7.73) | 4.7 (3.34, 6.16) | 1.3 (0.00, 2.73) |  | 8.5 (7.05, 9.87) | 8.9 (7.45, 10.3) | 2.2 (0.78, 3.60) |
| Na | 1.2 (1.02, 1.44) | 1.0 (0.87, 1.22) | 1.1 (0.95, 1.34) |  | 3.3 (2.78, 3.91) | 2.1 (1.75, 2.47) | 1.3 (1.11, 1.56) |
| P | 4.2 (3.55, 4.76) | 3.6 (2.97, 4.18) | 4.3 (3.71, 4.92) |  | 3.2 (2.56, 3.77) | 2.6 (2.04, 3.25) | 3.9 (3.33, 4.54) |
| S | 6.0 (4.86, 7.19) | 5.7 (4.52, 6.85) | 3.7 (2.57, 4.90) |  | 6.7 (5.54, 7.87) | 9.2 (8.00, 10.3) | 3.2 (2.00, 4.32) |
