# Supplementary material for: Fertilizer produced from abattoir waste can contribute to phosphorus sustainability, and biofortify crops with minerals
Source: PLoS One. 2019 Sep 4;14(9):e0221647. doi: 10.1371/journal.pone.0221647 (PMC6726140; doi:10.1371/journal.pone.0221647)
Supplement: S2 Table — Values in parentheses are the lower and upper confidence intervals, calculated at 95%. Only elements with measured concentrations above the limit of detection are shown, therefore for Co, Mo, Se, and Ti there are only data for the first cut. (DOCX) [file pone.0221647.s002.docx]

| Table S2a | Cut 1 | | | | | | | | |
| --- | --- | --- | --- | --- | --- | --- | --- | --- | --- |
|  |  |  | Optimal | | |  | Excess | | |
|  | Nil |  | NPK | Slow release | Thallo |  | NPK | Slow release | Thallo |
|  | mg/kg DM |  | mg/kg DM | | |  | mg/kg DM | | |
| Al | 170 (75.9, 375) |  | 90 (40.4, 199) | 130 (59.9, 296) | 150 (69.1, 341) |  | 180 (79.7, 393) | 120 (51.9, 256) | 80 (36.2, 178) |
| As | 0.54 (2.69, 0.815) |  | 0.39 (0.118, 0.664) | 0.43 (0.156, 0.702) | 0.53 (0.255, 0.801) |  | 0.90 (0.630, 1.18) | 0.36 (0.0908, 0.637) | 0.45 (0.173, 0.719) |
| Co | 0.086 (0.0554, 0.135) |  | 0.086 (0.0551, 0.135) | 0.045 (0.0289, 0.0706) | 0.089 (0.0572, 0.140) |  | 0.102 (0.0654, 0.160) | 0.092 (0.0591, 0.144) | 0.042 (0.0269, 0.0657) |
| Cr | 4.8 (3.53, 6.01) |  | 3.9 (2.62, 5.10) | 4.3 (3.1, 5.58) | 4.5 (3.22, 5.70) |  | 4.6 (3.34, 5.82) | 6.0 (4.72, 7.20) | 2.9 (1.65, 4.13) |
| Cu | 6.9 (5.56, 8.24) |  | 7.8 (6.47, 9.15) | 7.4 (6.1, 8.78) | 8.7 (7.38, 10.1) |  | 9.6 (8.22, 10.9) | 8.3 (6.93, 9.61) | 8.9 (7.57, 10.3) |
| Fe | 740 (341, 1620) |  | 510 (235, 1120) | 360 (164, 781) | 490 (225, 1070) |  | 340 (155, 739) | 490 (226, 1080) | 280 (128, 609) |
| Mn | 140 (97.6, 173) |  | 160 (123, 198) | 140 (106, 181) | 130 (93.0, 168) |  | 180 (140, 216) | 140 (103, 178) | 100 (64.5, 140) |
| Mo | 0.25 (0.159, 0.381) |  | 0.19 (0.122, 0.292) | 0.14 (0.087, 0.209) | 0.36 (0.233, 0.558) |  | 0.19 (0.121, 0.289) | 0.26 (0.165, 0.395) | 0.078 (0.0505, 0.121) |
| Ni | 8.3 (6.23, 11.1) |  | 7.5 (5.59, 9.96) | 4.2 (3.16, 5.63) | 9.6 (7.20, 12.8) |  | 5.1 (3.82, 6.81) | 5.8 (4.31, 7.68) | 5.2 (3.90, 6.96) |
| Se | 0.33 (0.190, 0.516) |  | 0.26 (0.139, 0.430) | 0.033 (0.00159, 0.103) | 0.48 (0.308, 0.701) |  | 0.0029 (0.00791, 0.0379) | 0.072 (0.0164, 0.168) | 0.57 (0.377, 0.804) |
| Ti | 1.1 (0.703, 1.63) |  | 0.80 (0.524, 1.22) | 1.2 (0.805, 1.87) | 1.9 (1.25, 2.90) |  | 1.8 (1.20, 2.78) | 0.90 (0.589, 1.37) | 0.75 (0.494, 1.15) |
| Zn | 32 (29.5, 35.0) |  | 32 (28.9, 34.4) | 32 (29.2, 34.7) | 37 (34.4, 39.9) |  | 36 (33.1, 38.6) | 32 (28.9, 34.4) | 38 (35.2, 40.7) |
|  | g/kg DM |  | g/kg DM | | |  | g/kg DM | | |
| Ca | 13 (11.7, 24.7) |  | 10 (9.07, 19.4) | 11 (10.0, 21.3) | 12 (11.0, 23.2) |  | 9.6 (8.41, 18.1) | 9.3 (8.09, 17.4) | 10 (9.44, 20.1) |
| K | 16 (14.8, 18.1) |  | 17 (14.9, 18.2) | 16 (14.5, 17.8) | 14 (12.6, 16.0) |  | 20 (18.2, 21.5) | 20 (18.0, 21.4) | 16 (14.0, 17.4) |
| Mg | 1.9 (1.7, 2.16) |  | 2.6 (2.40, 2.86) | 2.7 (2.47, 2.93) | 2.1 (1.83, 2.28) |  | 3.6 (3.33, 3.79) | 3.0 (2.81, 3.27) | 1.9 (1.70, 2.16) |
| Na | 1.8 (1.46, 2.12) |  | 2.0 (1.64, 2.30) | 2.1 (1.76, 2.43) | 1.9 (1.56, 2.23) |  | 3.0 (2.68, 3.35) | 2.1 (1.76, 2.32) | 1.9 (1.60, 2.26) |
| P | 1.5 (1.39, 1.56) |  | 1.8 (1.69, 1.91) | 1.9 (1.76, 1.98) | 1.8 (1.67, 1.89) |  | 2.1 (2.02, 2.28) | 2.1 (2.02, 2.28) | 2.2 (2.05, 2.31) |
| S | 2.2 (1.79, 2.71) |  | 3.1 (2.69, 3.60) | 3.2 (2.75, 3.67) | 3.2 (2.75, 3.67) |  | 3.4 (2.99, 3.91) | 3.7 (3.20, 4.12) | 3.2 (2.73, 3.65) |

| Table S2b | Cut 2 | | | | | | | | |
| --- | --- | --- | --- | --- | --- | --- | --- | --- | --- |
|  |  |  | Optimal | | |  | Excess | | |
|  | Nil |  | NPK | Slow release | Thallo |  | NPK | Slow release | Thallo |
|  | mg/kg DM |  | mg/kg DM | | |  | mg/kg DM | | |
| Al | 15 (6.74, 33.2) |  | 13 (5.92, 29.2) | 10 (4.68, 23.1) | 25 (11.1, 54.8) |  | 14 (6.30, 31.1) | 15 (6.52, 32.2) | 16 (7.39, 36.4) |
| As | 0.28 (0.00378, 0.550) |  | 0.34 (0.638, 0.610) | 0.52 (2.44, 0.790) | 0.49 (2.12, 0.758) |  | 0.30 (0.0288, 0.575) | 0.06 (0, 0.332) | 0.24 (0, 0.516) |
| Co |  |  |  |  |  |  |  |  |  |
| Cr | 3.5 (2.23, 4.71) |  | 2.7 (1.45 (3.93) | 2.5 (1.24, 3.72) | 2.1 (0.886, 3.37) |  | 1.9 (0.606, 3.09) | 1.7 (0.476, 2.96) | 2.1 (0.866, 3.35) |
| Cu | 4.5 (3.11, 5.79) |  | 4.3 (2.96, 5.64) | 4.2 (2.83, 5.51) | 4.6 (3.3, 5.98) |  | 5.0 (3.67, 6.35) | 4.4 (3.05, 5.73) | 5.4 (4.08, 6.76) |
| Fe | 41 (19.0, 90.3) |  | 36 (16.3, 77.6) | 38 (17.6, 83.7) | 48 (22.0, 105) |  | 35 (16.1, 76.9) | 29 (13.2, 63.1) | 50 (22.8, 108) |
| Mn | 330 (290, 366) |  | 430 (387, 462) | 420 (385, 461) | 390 (349, 424) |  | 420 (385, 460) | 410 (376, 452) | 330 (296, 371) |
| Mo |  |  |  |  |  |  |  |  |  |
| Ni | 5.7 (4.27, 7.61) |  | 4.5 (3.41, 6.07) | 4.4 (3.33, 5.93) | 5.1 (3.80, 6.77) |  | 3.7 (2.81, 5.01) | 3.8 (2.82, 5.03) | 4.8 (3.63, 6.46) |
| Se |  |  |  |  |  |  |  |  |  |
| Ti |  |  |  |  |  |  |  |  |  |
| Zn | 26 (23.5, 29.0) |  | 25 (22.6, 28.0) | 26 (23.3, 28.8) | 28 (25.2, 30.7) |  | 25 (22.6, 28.1) | 26 (22.8, 28.3) | 30 (27.2, 32.6) |
|  | g/kg DM |  | g/kg DM | | |  | g/kg DM | | |
| Ca | 7.6 (6.40, 14.0) |  | 7.8 (6.53, 14.3) | 7.6 (6.41, 14.0) | 9.2 (7.99, 17.2) |  | 8.1 (6.87, 15.0) | 7.0 (5.82, 12.9) | 11 (9.79, 20.8) |
| K | 14 (12.1, 15.4) |  | 11 (9.54, 12.9) | 12 (10.3, 13.6) | 11 (9.21, 12.5) |  | 8.4 (6.69, 10.0) | 11 (9.23, 12.6) | 10 (7.90, 11.2) |
| Mg | 2.7 (2.48, 2.94) |  | 3.5 (3.24, 3.70) | 3.5 (3.28, 3.74) | 2.8 (2.61, 3.07) |  | 4.1 (3.85, 4.30) | 4.0 (3.79, 4.25) | 2.8 (2.60, 3.06) |
| Na | 0.77 (0.434, 1.10) |  | 0.86 (0.532, 1.19) | 0.84 (0.505, 1.17) | 0.67 (0.335, 1.00) |  | 1.0 (0.649, 1.31) | 1.0 (0.645, 1.31) | 0.65 (0.316, 0.978) |
| P | 2.2 (2.09, 2.35) |  | 1.8 (1.72, 1.94) | 1.9 (1.78, 2.01) | 1.9 (1.76, 1.99) |  | 1.9 (1.83, 2.06) | 2.0 (1.91, 2.16) | 2.1 (1.94, 2.19) |
| S | 2.5 (2.00, 2.92) |  | 5.7 (5.21, 6.13) | 5.7 (5.21, 6.13) | 5.6 (5.19, 6.11) |  | 5.5 (5.01, 5.92) | 5.9 (5.43, 6.35) | 6.0 (5.54, 6.46) |

| Table S2c | Cut 3 | | | | | | | | |
| --- | --- | --- | --- | --- | --- | --- | --- | --- | --- |
|  |  |  | Optimal | | |  | Excess | | |
|  | Nil |  | NPK | Slow release | Thallo |  | NPK | Slow release | Thallo |
|  | mg/kg DM |  | mg/kg DM | | |  | mg/kg DM | | |
| Al | 75 (33.7, 166) |  | 40 (18.0, 88.8) | 41 (18.3, 90.5) | 20 (9.11, 44.9) |  | 130 (56.7, 280) | 38 (17.2, 85.0) | 44 (19.8, 97.6) |
| As | 0.57 (0.293, 0.839) |  | 0.14 (0, 0.413) | 0.42 (0.142, 0.688) | 0.16 (0, 0.436) |  | 0.19 (0, 0.458) | 0.38 (0.106, 0.652) | 0.17 (0, 0.441) |
| Co |  |  |  |  |  |  |  |  |  |
| Cr | 1.9 (0.686, 3.17) |  | 1.3 (0.096, 2.58) | 2.1 (0.846, 3.33) | 1.6 (0.346, 2.83) |  | 1.7 (0.486, 2.97) | 3.6 (2.31, 4.79) | 1.8 (0.576, 3.06) |
| Cu | 7.9 (6.60, 9.28) |  | 7.2 (5.89, 8.57) | 6.2 (4.90, 7.58) | 6.9 (5.51, 8.19) |  | 9.1 (7.79, 10.5) | 6.5 (5.18, 7.86) | 6.7 (5.33, 8.01) |
| Fe | 410 (186, 887) |  | 54 (24.8, 118) | 110 (51.0, 243) | 170 (78.0, 371) |  | 110 (51.9, 247) | 110 (51.9, 247) | 88 (40.3, 192) |
| Mn | 310 (274, 350) |  | 430 (392, 468) | 440 (404, 479) | 380 (339, 415) |  | 530 (490, 565) | 460 (420, 495) | 390 (356, 431) |
| Mo |  |  |  |  |  |  |  |  |  |
| Ni | 9.7 (7.29, 13.0) |  | 8.1 (6.05, 10.8) | 7.7 (5.80, 10.3) | 9.6 (7.17, 12.8) |  | 7.5 (5.63, 10.0) | 7.4 (5.58, 9.94) | 9.0 (6.75, 12.0) |
| Se |  |  |  |  |  |  |  |  |  |
| Ti |  |  |  |  |  |  |  |  |  |
| Zn | 32 (29.4, 34.9) |  | 31 (28.0, 33.5) | 32 (29.6, 35.1) | 35 (32.0, 37.5) |  | 32 (29.5, 35.0) | 34 (31.1, 36.6) | 37 (34.6, 40.1) |
|  | g/kg DM |  | g/kg DM | | |  | g/kg DM | | |
| Ca | 7.8 (6.55, 14.3) |  | 7.5 (6.30, 13.8) | 7.4 (6.17, 13.6) | 8.2 (6.95, 15.1) |  | 7.1 (5.86, 13.0) | 6.5 (5.29, 11.8) | 9.4 (8.13, 17.5) |
| K | 17 (15.4, 18.7) |  | 22 (20.5, 23.9) | 22 (20.3, 23.6) | 21 (19.8, 23.1) |  | 22 (19.9, 23.3) | 22 (20.4, 23.8) | 21 (19.0, 22.4) |
| Mg | 2.9 (2.65, 3.10) |  | 4.0 (3.79, 4.25) | 3.9 (3.67, 4.12) | 3.1 (2.91, 3.37) |  | 4.8 (4.54, 5.00) | 4.9 (4.63, 5.09) | 3.4 (3.16, 3.62) |
| Na | 1.4 (1.12, 1.78) |  | 1.3 (0.92, 1.58) | 1.5 (1.20, 1.86) | 1.3 (0.946, 1.61) |  | 1.1 (0.794, 1.46) | 1.6 (1.24, 1.91) | 1.1 (0.775, 1.44) |
| P | 4.3 (4.00, 4.52) |  | 4.2 (3.91, 4.41) | 4.2 (3.91, 4.41) | 4.1 (3.82, 4.31) |  | 3.8 (3.58, 4.04) | 4.2 (3.92, 4.42) | 3.9 (3.66, 4.13) |
| S | 2.8 (2.39, 3.31) |  | 7.5 (6.99, 7.91) | 7.3 (6.81, 7.73) | 6.7 (6.23, 7.15) |  | 8.0 (7.54, 8.46) | 8.3 (7.82, 8.74) | 7.9 (7.47, 8.39) |
