# Supplementary material for: Fertilizer produced from abattoir waste can contribute to phosphorus sustainability, and biofortify crops with minerals
Source: PLoS One. 2019 Sep 4;14(9):e0221647. doi: 10.1371/journal.pone.0221647 (PMC6726140; doi:10.1371/journal.pone.0221647)
Supplement: S3 Table — Values in parentheses are the lower and upper confidence intervals, calculated at 95%. Only elements with measured concentrations above the limit of detection are shown. (DOCX) [file pone.0221647.s003.docx]

|  |  |  | Optimal | | |  | Excess | | |
| --- | --- | --- | --- | --- | --- | --- | --- | --- | --- |
|  | Nil |  | NPK | Slow release | Thallo |  | NPK | Slow release | Thallo |
|  | µg/kg DM |  | µg/kg DM | | |  | µg/kg DM | | |
| Co | 340 (47.1, 2440) |  | 0.20 (0.114, 0.821) | 0.20 (0.114, 0.821) | 0.20 (0.114, 0.821) |  | 11 1.55, 75.6) | 0.58 (0.166, 3.551) | 0.66 (0.178, 4.16) |
| Mo | 1410 (165, 12100) |  | 7.9 (1.01, 66.6) | 23 (2.77, 196.5) | 53 (6.22, 450) |  | 3.3 (0.478, 27.8) | 0.70 (0.170, 5.27) | 39 (4.64, 334) |
| Pb | 170 (80.1, 254) |  | 29 (0, 116) | 9.0 (0, 95.9) | 45 (0, 132) |  | 0 (0, 66.9) | 0 (0, 81.9) | 17 (0, 104) |
| Ti | 920 (463, 1840) |  | 52 (26.1, 104) | 65 (32.8, 130) | 119 (59.6, 236) |  | 133 (67.0, 265) | 95 (47.5, 188) | 67 (33.7, 134) |
|  | mg/kg DM |  | mg/kg DM | | |  | mg/kg DM | | |
| Al | 27 16.3, 44.4) |  | 2.8 (1.71, 4.65) | 2.5 (1.50, 4.08) | 2.6 (1.60, 4.37) |  | 3.8 (2.28, 6.20) | 3.7 (2.22, 6.06) | 3.1 (1.85, 5.04) |
| As | 0.34 (0.128, 0.546) |  | 0.14 (0, 0.353) | 0.22 (0.00586, 0.424) | 0.16 (0, 0.369) |  | 0.17 (0, 0.377) | 0.18 (0, 0.384) | 0.13 (0, 0.340) |
| Ca | 50 (464, 526) |  | 38 (351, 413) | 37 (334, 396) | 49 (457, 519) |  | 35 (317, 380) | 36 (327, 389) | 70 (669, 731) |
| Cd | 0.0543 (0.0542, 0.0544) |  | 0.178 (0.177, 0.178) | 0.177 (0.177, 0.177) | 0.0931 (0.0930, 0.0932) |  | 0.134 (0.134, 0.134) | 0.210 (0.210, 0.210) | 0.0820 (0.0819, 0.0821) |
| Cr | 21 (9.20, 49.7) |  | 0.25 (0.106, 0.575) | 0.28 (0.120, 0.648) | 0.28 (0.119, 0.645) |  | 0.32 (0.139, 0.751) | 0.28 (0.122, 0.658) | 0.47 (0.204, 1.10) |
| Cu | 9.3 (8.13, 10.7) |  | 3.4 (2.99, 3.95) | 4.1 (3.59, 4.73) | 3.7 (3.23, 4.26) |  | 3.8 (3.28, 4.33) | 4.3 (3.76, 4.96) | 5.1 (4.43, 5.84) |
| Fe | 1300 (719, 2360) |  | 31 (17.4, 57.0) | 28 (15.7, 51.5) | 29 (16.1, 52.7) |  | 49 (27.2, 89.3) | 42 (23.2, 76.2) | 49 (27.2, 89.1) |
| Mn | 150 (127, 167) |  | 90 (78.4, 103) | 99 (86.7, 114) | 75 (65.5, 85.9) |  | 80 (69.7, 91.4) | 93 (81.1, 106) | 63 (55.2, 72.4) |
| Na | 17 (13.4, 21.0) |  | 9.4 (7.53, 11.7) | 10 (8.05, 12.5) | 8.4 (6.72, 10.5) |  | 14 (11.6, 18.1) | 12 (9.43, 14.7) | 13 (10.2, 15.8) |
| Ni | 11 (8.58, 15.0) |  | 0.92 (0.70, 1.22) | 0.76 (0.58, 1.01) | 0.76 (0.57, 1.00) |  | 0.98 (0.74, 1.29) | 0.88 (0.66, 1.16) | 0.93 (0.70, 1.23) |
| Se | Below LOD |  | 0.82 (0, 0.180) | 0 (0, 0.0978) | 0.47 (0.371, 0.567) |  | 0.22 (0.120, 0.316) | 0.011 (0, 0.109) | 0.66 (0.558, 0.754) |
| Zn | 73 (67.0, 78.3) |  | 51 (45.0, 56.3) | 48 (42.1, 53.4) | 55 (49.6, 60.9) |  | 58 (52.7, 63.9) | 58 (52.7, 64.0) | 86 (80.4, 91.6) |
|  | g/kg DM |  | g/kg DM | | |  | g/kg DM | | |
| K | 5.6 (5.26, 5.91) |  | 4.6 (4.28, 4.93) | 4.6 (4.31, 4.96) | 4.3 (4.02, 4.67) |  | 5.7 (5.41, 6.06) | 5.4 (5.08, 5.73) | 5.4 (5.04, 5.69) |
| Mg | 1.9 (1.87, 2.03) |  | 1.4 (1.31, 1.47) | 1.4 (1.36, 1.51) | 1.2 (1.09, 1.24) |  | 1.7 (1.63, 1.78) | 1.7 (1.61, 1.76) | 1.3 (1.21, 1.37) |
| P | 5.4 (5.29, 5.57) |  | 3.6 (3.42, 3.71) | 3.8 (3.63, 3.92) | 3.4 (3.27, 3.56) |  | 4.3 (4.15, 4.43) | 4.5 (4.31, 4.59) | 4.2 (4.08, 4.37) |
| S | 1.5 (1.42, 1.62) |  | 1.4 (1.34, 1.54) | 1.4 (1.26, 1.46) | 1.3 (1.22, 1.41) |  | 1.7 (1.62, 1.81) | 1.6 (1.54, 1.74) | 1.8 (1.72, 1.91) |
