# Supplementary material for: Fertilizer produced from abattoir waste can contribute to phosphorus sustainability, and biofortify crops with minerals
Source: PLoS One. 2019 Sep 4;14(9):e0221647. doi: 10.1371/journal.pone.0221647 (PMC6726140; doi:10.1371/journal.pone.0221647)
Supplement: S4 Table — Values in parentheses are the lower and upper confidence intervals, calculated at 95%. Only elements with measured concentrations above the limit of detection are shown. (DOCX) [file pone.0221647.s004.docx]

|  |  |  | Optimal | | |  | Excess | | |
| --- | --- | --- | --- | --- | --- | --- | --- | --- | --- |
|  | Nil |  | NPK | Slow release | Thallo |  | NPK | Slow release | Thallo |
|  | mg/kg DM |  | mg/kg DM | | |  | mg/kg DM | | |
| Al | 174 (144, 203) |  | 42 (12.5, 71.9) | 33 (2.93, 62.3) | 55 (25.4, 84.8) |  | 40 (10.2, 69.6) | 56 (26.5, 85.9) | 39 (9.73, 69.1) |
| As | 0.31 (0.0896, 0.520) |  | 0.32 (0.100, 0.530) | 0.22 (0.00664, 0.437) | 0.39 (0.172, 0.602) |  | 0.50 (0.286, 0.716) | 0.028 (0, 0.243) | 0.32 (0.104, 0.534) |
| Cd | 0.051 (0.0215, 0.0801) |  | 0.37 (0.336, 0.394) | 0.35 (0.321, 0.380) | 0.20 (0.166, 0.225) |  | 0.31 (0.281, 0.340) | 0.43 (0.403, 0.462) | 0.19 (0.162, 0.221) |
| Cr | 5.5 (3.11, 9.81) |  | 2.4 (1.38, 4.34) | 1.6 (0.888, 2.80) | 3.4 (1.92, 6.08) |  | 1.8 (1.04, 3.28) | 3.4 (1.89, 5.98) | 2.3 (1.28, 4.03) |
| Cu | 2.3 (2.04, 2.54) |  | 1.9 (1.69, 2.18) | 1.9 (1.61, 2.10) | 1.9 (1.65, 2.14) |  | 3.0 (2.74, 3.24) | 2.5 (2.28, 2.78) | 3.3 (3.05, 3.54) |
| Fe | 1100 (942, 1190) |  | 54 (0, 177) | 43 (0, 166) | 88 (0, 211) |  | 57 (0, 180) | 91 (0, 214) | 180 (57.3, 303) |
| Mn | 230 (195, 271) |  | 320 (285, 360) | 320 (282, 357) | 210 (175, 251) |  | 320 (282, 357) | 310 (270, 345) | 150 (109, 184) |
| Na | 110 (59.8, 163) |  | 150 (97.2, 201) | 230 (176, 279) | 70 (18.2, 122) |  | 300 (247, 351) | 260 (212, 316) | 290 (236, 339) |
| Ni | 2.6 (1.45, 4.77) |  | 1.3 (0.744, 2.44) | 0.83 (0.458, 1.50) | 1.9 (1.03, 3.40) |  | 1.2 (0.676, 2.22) | 1.6 (0.857, 2.81) | 1.6 (0.862, 2.83) |
| Ti | 2.2 (0.928, 5.35) |  | 0.36 (0.150, 0.864) | 0.27 (0.114, 0.657) | 0.33 (0.138, 0.795) |  | 0.33 (0.137, 0.791) | 0.45 (0.187, 1.07) | 0.37 (0.154, 0.886) |
| Zn | 13 (8.77, 18.0) |  | 29 (24.4, 33.6) | 25 (20.2, 29.4) | 38 (33.3, 42.5) |  | 30 (25.4, 34.6) | 27 (22.4, 31.6) | 61 (56.0, 65.2) |
|  | g/kg DM |  | g/kg DM | | |  | g/kg DM | | |
| Ca | 1.8 (1.33, 2.33) |  | 4.5 (3.95, 4.95) | 3.5 (3.02, 4.02) | 6.0 (5.50, 6.51) |  | 3.9 (3.37, 4.37) | 3.7 (3.16, 4.17) | 9.7 (9.22, 10.2) |
| K | 17 (15.1, 18.7) |  | 20 (18.3, 21.9) | 19 (17.5, 21.1) | 17 (14.7, 18.3) |  | 24 (22.4, 26.0) | 24 (21.9, 25.5) | 17 (15.7, 19.3) |
| Mg | 0.62 (0.552, 0.686) |  | 3.0 (2.71, 3.37) | 2.5 (2.28, 2.84) | 1.4 (1.22, 1.52) |  | 4.6 (4.10, 5.10) | 4.5 (4.03, 5.01) | 1.9 (1.69, 2.11) |
| P | 0.82 (0.621, 1.01) |  | 0.59 (0.391, 0.785) | 0.94 (0.745, 1.14) | 0.55 (0.357, 0.751) |  | 1.5 (1.28, 1.68) | 1.7 (1.50, 1.90) | 1.4 (1.17, 1.57) |
| S | 0.65 (0.575, 0.745) |  | 4.1 (3.57, 4.63) | 4.1 (3.58, 4.64) | 3.2 (2.78, 3.60) |  | 4.6 (4.06, 5.26) | 5.4 (4.70, 6.09) | 4.7 (4.13, 5.35) |
